# Schistosomiasis in immigrants, refugees and travellers in an Italian referral centre for tropical diseases

**DOI:** 10.1186/s40249-018-0440-5

**Published:** 2018-06-16

**Authors:** Valentina Marchese, Anna Beltrame, Andrea Angheben, Geraldo Badona Monteiro, Giovanni Giorli, Francesca Perandin, Dora Buonfrate, Zeno Bisoffi

**Affiliations:** 1 Centre for Tropical Diseases, Sacro Cuore Don Calabria Hospital, Via Sempreboni 5, 37024 Negrar, Italy; 2 University Department of Infectious and Tropical Diseases & WHO Collaborating Centre for TB/HIV and TB elimination, University of Brescia, Piazzale Spedali Civili 1, 25123 Brescia, Italy

**Keywords:** Schistosomiasis, Neglected tropical diseases, immigrants, Refugees, Travellers, Europe, Italy

## Abstract

**Background:**

Schistosomiasis is one of the most important neglected tropical diseases. If unrecognised and untreated, the chronic infection can lead to irreversible complications.

**Methods:**

Retrospective observational study aimed at describing clinical history, laboratory findings and imaging presentation of imported schistosomiasis diagnosed at the Centre for Tropical Diseases, Sacro Cuore Don Calabria Hospital of Negrar, Verona, Italy from 2010 to 2014. The aim of our study was to assess differences in demographic characteristics, clinical presentation, laboratory data and ultrasound findings between immigrants/visiting friends and relatives (VFR) from endemic countries (endemic group) and expatriates/travellers (non-endemic group).

**Results:**

A total of 272 patients were retrieved: 234 in the endemic and 38 in the non-endemic group. Most of the patients acquired schistosomiasis in Africa (97.4%). Symptoms were reported by 52.9% of the patients; abdominal pain (36%), macroscopic hematuria (11.3%), and genito-urinary symptoms (7.4%) being the most frequently reported. Increased IgE and blood eosinophilia were observed in 169 (63.8%) and 130 (47.8%) patients, respectively. The proportion of positive serology was 250/272 (91.9%).The Circulating Cathodic Antigen CCA for *Schistosoma mansoni* was positive in 14/61 individuals (23%). At microscopy, infected subjects were 103/272 (37.9%). The species of Schistosoma found were *S. haematobium* (47.6%), *S. mansoni* (46.6%) or both (5.8%). Schistosomiasis was classified as confirmed in 103 (37.9%), probable in 165 (60.6%) and suspected in 4 (1.5%) cases using clinical presentation, laboratory data and ultrasound findings. The infection was further classified based on organ involvement: intestinal (17.9%), hepatosplenic (5.1%), urogenital (48.9%), and indeterminate (43.8%).

The comparative analysis of endemic and non-endemic patients highlighted differences in sex and age. Endemic patients had more frequent ova identification (41.9% vs. 13.2%, *P* < 0.001) and increased IgE (70% vs. 26.3%, *P* < 0.001) when compared with non-endemic. Multivariate analyses showed that younger age, abnormal ultrasound findings and blood eosinophilia were significantly associated with positive microscopy (*OR* = 0.94, *OR* = 2.12, *OR* = 1.98, respectively).

**Conclusions:**

Symptoms, eosinophilia and abnormal ultrasound findings were present in about half of patients, without differences between groups. Many patients had positive serology but negative microscopy, indicating that schistosomiasis might be misdiagnosed. A combination of diagnostic tools may facilitate the diagnosis.

**Electronic supplementary material:**

The online version of this article (10.1186/s40249-018-0440-5) contains supplementary material, which is available to authorized users.

## Multilingual abstracts

Please see Additional file 1 for translations of the abstract into the five official working languages of the United Nations.

## Background

Schistosomiasis is one of the most important neglected tropical diseases (NTDs), affecting over 258 million people in 78 countries and causing 3.3 million disability-adjusted life-years (DALYs) [1–4]. In sub-Saharan Africa, where around 90% of the global cases occur, approximately 300 000 deaths annually have been estimated to be caused by schistosomiasis [5].

*Schistosoma mansoni* and *S. haematobium*, the predominating human species, cause intestinal schistosomiasis (IS), hepatosplenic schistosomiasis (HSS) and urogenital schistosomiasis (UGS), respectively [1, 6]. Infections caused by *S. intercalatum*, *S. japonicum* and *S. mekongi* are rarely reported, the latter two restricted to Asia [7].

People born in endemic countries are at high risk of acquiring the infection during childhood, by contact with contaminated freshwater. The exposure continues in later years during domestic, recreational and economic activities [1, 6]. As the lifetime of the adult *Schistosoma* ranges from three to 40 years [8, 9], chronic *Schistosoma* infections may remain clinically silent (30–50% of the cases) [10] or cause relatively mild and non-specific symptoms for years before eventually causing severe, often unrecoverable organ damage such as fibrosis and portal hypertension for *S. mansoni*, and obstructive uropathy and hydronephrosis for *S. haematobium* [11–13]. Interestingly, the latter may lead to squamous cell carcinoma of the bladder [14, 15]. However, as clearly highlighted by King [16], schistosomiasis should never be considered a benign asymptomatic infection, as it always leads to chronic tissue inflammation when left untreated. Unfortunately, low-intensity infections may be easily missed by the most widely available diagnostic test, that is microscopy.

Europe, and Italy in particular, is receiving an unprecedented flow of immigrants from sub-Saharan Africa, many asylum seekers [17]. According to GeoSentinel [a network of 41 tropical and travel medicine centres in 19 countries around the world (http://www.istm.org/geosentinel)] from 1997 to 2009, schistosomiasis was diagnosed in 442 of 7629 (5.8%) migrants, predominantly non-refugees, 84% of whom acquired the infection in Africa [18]. In these last years, schistosomiasis prevalence among African refugees and asylum seekers has been striking, albeit mostly unrecognized as sensitive screening tools are generally unavailable [19–26].

Sub-Saharan Africa is a frequent destination reported by European travellers [27]. In the EuroTravNet network of 18 European specialized centres (http://www.eurotravnet.eu), which reported the travel-related illnesses of 32136 travellers from 2008 to 2012, schistosomiasis ranked 12th with only 152 cases recorded [27]. Nevertheless, the risk of schistosomiasis is not negligible in this group; often after a single brief exposure to contaminated freshwater [28, 29]. Several cases series of schistosomiasis in European travellers have been reported in recent years [30–36].

An unexpected outbreak of autochthonous urogenital schistosomiasis cases occurred in Corsica, France during the period 2013–2015 [37, 38], caused by a parasite genetically related to *S. haematobium* from Senegal [39, 40]. The outbreak raised attention to a possible spread of schistosomiasis in areas of Southern Europe [41–43] where the intermediate host is present [44]. Hence, surveillance on imported schistosomiasis in Europe, together with an increased professional awareness for this NTD, are necessary [45].

This study reviews demographic data, travel history, clinical and laboratory information, imaging and other diagnostic findings of the largest case series of imported schistosomiasis observed in a single site (the Centre for Tropical Diseases, [CTD] Sacro Cuore Don Calabria Hospital, Negrar, Verona) in Italy over a 5-year period.

## Methods

### Study design

Retrospective, observational study intended to review patients diagnosed with schistosomiasis at CTD and to assess differences between subjects born in endemic and non-endemic countries.

### Study population and data collection

The study was carried out at the CTD, a national referral centre for tropical and parasitic diseases, between 1 January 2010 and 31 December 2014. Patients were included in the study if they met diagnostic criteria for schistosomiasis, described in the case definition paragraph. All patients were treated with praziquantel 40 mg/kg for three consecutive days. Patients lacking a written informed consent for collection of data and biological samples for study purposes were excluded from the study.

For each included patient, we reviewed from a CTD patient database the travel history, clinical and laboratory data, imaging, and other diagnostic procedure findings. Data were analysed anonymously.

The study subjects were classified into two groups:

**Endemic group**: immigrants from countries where schistosomiasis is endemic, differentiated into either immigrants, refugees or asylum seekers recently arrived in Italy, or non-recent immigrants who had travelled to their countries of origin for business purpose or visiting friends or relatives (VFR);

**Non-endemic group**: Italians visiting endemic countries, including expatriates and short-term travellers (tourists, students, business travellers).

For the endemic group, the country of origin was recorded as the probable area of exposure. The period of time going from the date of first arrival in Italy to the date of diagnosis was defined as “elapsed time”. For the non-endemic group, the country visited and the elapsed time (days from the date of return to the date of diagnosis) were recorded. If the patient reported possible exposure in more than one endemic area for schistosomiasis, the country of the longer exposure was selected, unless there were clear indications for acquisition of the infection in a different country.

The symptoms and signs included were abdominal pain, defined as non-specific abdominal discomfort and pain, and genito-urinary symptoms, comprehensive of urinary frequency, dysuria and/or burning and/or painful ejaculation. Additionally, Katayama syndrome, cough and gynaecological/andrological complaints were classified as other symptoms, while the presence of blood in urine or stool was recorded as macrohematuria or rectal bleeding, respectively.

### Laboratory data

The blood test evaluation of each patient included blood cell coun**t** and IgE quantitative nephelometry assay. Blood eosinophilia was defined in case of absolute eosinophil count (AEC) value ≥ 300 cell/μl and increased IgE if total IgE value equal or above the upper normal limit (≥ 120 IU/ml).

Serum samples were evaluated for the detection of antibodies against *Schistosoma spp.* by an indirect immunofluorescence (IFAT) using whole cercarial antigen (DiaTeSt-Cercaria FAT) from January 2010 to September 2012. An Enzyme-linked immunosorbent assay (ELISA) using *S. mansoni* soluble egg antigens (Bordier Affinity Products, Crissier, Switzerland) was introduced from February 2012 to December 2014. Other immunodiagnostic methods were used to detect the presence of *Strongyloides stercoralis* and viral infections: an in-house indirect immunofluorescence (IFAT) for *S. stercoralis* [46] and enzyme-linked immunosorbent assay (ELISA, Beckmann) for HIV, HCV and HBsAg with a confirmatory test in case of a positive result.

Finally, since March 2014, urine circulating cathodic antigen (CCA) dipstick test for *S. mansoni* was available: from a single urine sample a 50 μl aliquot was used to test for the presence of schistosoma CCA with a commercially available immuno-chromatographic dipstick test following the manufacturer’s instruction (NADAL CCA Bilharzia test, Germany).

Microscopy examination (100 ×) for ova and parasites, after formol-ether concentration on three stool samples collected in alternate days was performed for each patient together with an urine microscopy examination (100 ×) after micropore filtration for *S. haematobium* on the sediment of at least 20 ml end-stream urine. One to three urine samples were obtained from 10 am to 12 am over consecutive days. Microscopy detection of eggs in biopsy of any tissue specimen was also performed, if done.

### Ultrasounds and other diagnostic procedures

The results of abdomen ultrasounds and other invasive tests, such as liver biopsy, were recorded. In particular, an ultrasound finding was considered abnormal in the case of: liver or spleen enlargement (a rounding of the inferior liver margin, and bipolar spleen diameter > 12 cm), periportal thickening, cirrhosis, portal hypertension, bladder wall thickening or lesions (calcification or small parietal vegetation), an abnormality in the urinary tract (ureter dilatation or hydronephrosis) or lesions compatible with liver or bladder cancer [47–49].

Two abnormal ultrasound patterns that likely correlated to the schistosomiasis were recorded:

**Urogenital (UG) pattern**: bladder wall thickening or lesions, an abnormality of the urinary tract or lesions compatible with bladder cancer.

**Hepatosplenic (HS) pattern**: presence of periportal thickening, cirrhosis or portal hypertension.

### Case definition

Based on clinical, laboratory, and ultrasounds findings, we operationally classified schistosomiasis as [32, 37] confirmed schistosomiasis in case of retrieval of Schistosoma ova (either in stool, urine or specimen biopsy). Whenever a patient had positive urine CCA and/or serology test without evidence of ova, the case was classified as having probable schistosomiasis**.** A possible schistosomiasis was defined with abnormal ultrasound patterns (as defined previously) and/or total IgE ≥ 1000 IU/ml, without evidence of ova or with negative urine CCA or serology test [32].

Additionally, based on signs of organ involvement, we differentiated schistosomiasis into intestinal schistosomiasis (IS) if ova of *S. mansoni* or *S. mekongi* or *S. japonicum* were found in the stool or in the rectal biopsy. If ova of *S. mansoni* or *S. mekongi* or *S. japonicum* were found in the liver biopsy or if the ultrasounds revealed an HS pattern (as defined above) the case was considered as hepatosplenic schistosomiasis (HSS). Urogenital schistosomiasis (UGS) was diagnosed if ova of *S. haematobium* were found in the urine or bladder biopsy, if the patient reported macroscopic hematuria or genito-urinary symptoms or if the ultrasound revealed a UG pattern (as defined above). In absence of ova, positive serology and/or urine CCA test, and without any ultrasound patterns or specific symptoms, the patient was considered having indeterminate schistosomiasi (I).

### Statistical analysis

Descriptive statistics were used to analyse the characteristics of the entire cohort and then separately for individuals born in endemic areas and individuals born in Italy. Categorical variables were reported as frequencies and proportions, while quantitative variables were presented as median (inter-quartile range, IQR).

The association between the aforementioned variables was then investigated through univariate statistical tests, such as the Chi-squared test, Fisher test and Kolmogorov-Smirnov test, as considered appropriate. Lastly, multivariate logistic regression models were fitted to assess the impact of all covariates on the probability of contracting symptoms related to schistosomiasis and on the probability of detecting ova in any specimen. All statistical analyses were conducted using R, version 3.3.3 [50].

## Results

### Demographic data and travel history

Demographic data and travel-related information are detailed in Table 1. Of the 272 subjects, 215 (79%) were male and the median age was 30 (IQR:25–39.5). Most subjects (86%) were immigrants, of whom, 76.1% (178 subjects) were either refugees or asylum seekers, whereas in the non-endemic group (38 subjects) travellers and expatriates were equally represented (19 subjects in both sub-groups). The male gender was more represented in the immigrant group than in the travellers of the non-endemic group (82.5 vs 57.9%, *P* = 0.002). The median age of the immigrants was significantly lower than that of the patients in the non-endemic group (28 vs 41.5 years, *P* < 0.001).

**Table 1:** Baseline demographic of the cohort and stratified by endemicity of provenience

Characteristic^a^	Entire cohort	Area of origin		*p*-value^b^
	*N*, %	Endemic	Non-endemic	
Total	272	234 (86.0)	38 (14.0)	
Median age, years (IQR)	30 (25–39.5)	28 (24–38)	41.5 (34–63.7)	< 0.001*
Male, *N* (%)	215 (79.0)	193 (82.5)	22 (57.9)	0.002*
Median elapsed time, days (IQR)	197 (67.5–526.5)	209 (71–576.5)	120.5 (52–392.7)	0.327
Continent of Exposure, *N* (%)				
Africa	261 (97.4)	232 (99.1)	29 (85.3)	< 0.001*
America	4 (1.5)	2 (0.9)	2 (5.9)	0.089
Asia and Oceania	3 (1.1)	0 (0.0)	3 (8.8)	

*Abbreviations: IQR* Inter-quartile range

^a^Relative frequencies for each variable are obtained on the sub-population for which information was available

^b^Calculation of the *P*-value was done through Kolmogorov-Smirnov test for the difference in the distribution of age and elapsed time, whereas Fisher’s exact test was used for all dichotomous variables. Finally, the Chi-Squared test was used to test the difference in the distribution among diagnoses types

**Fig. 1:**
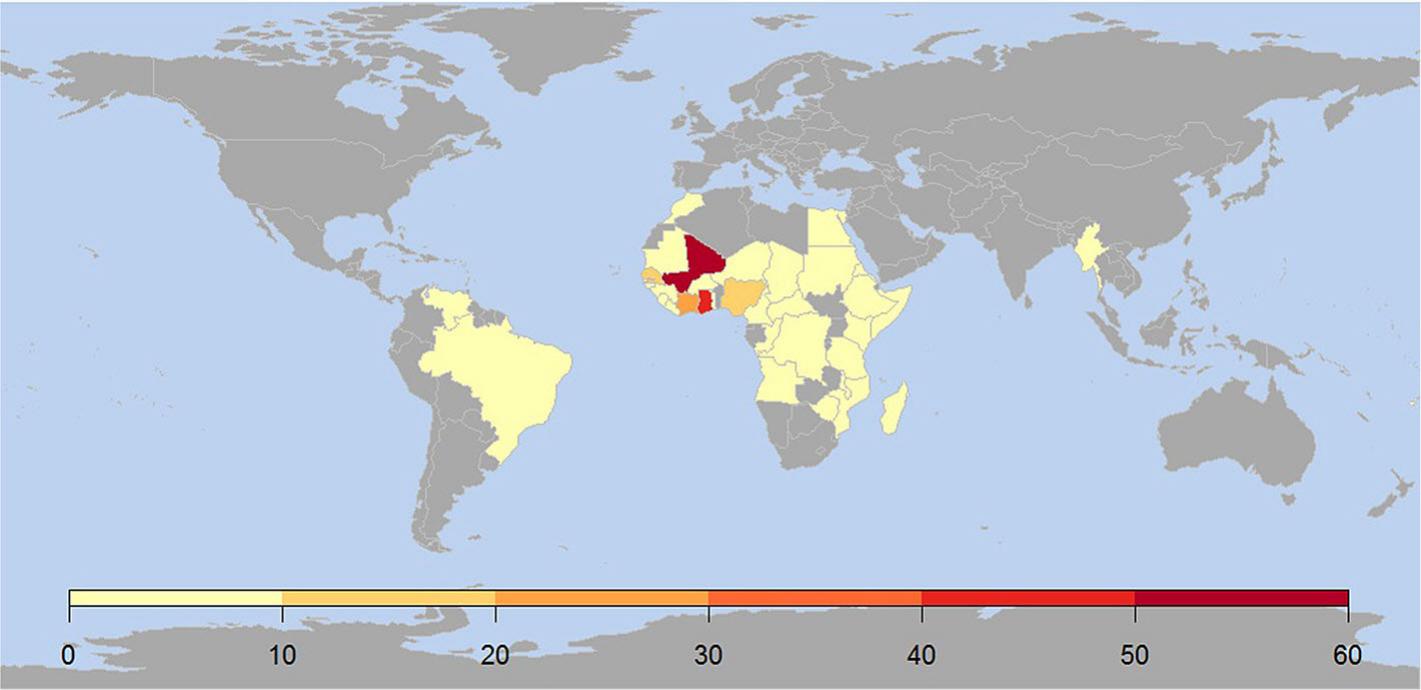
Geographical distribution of the places of exposure (data are the number of patients)

Almost all cases originated from Africa (*n* = 261, 97.4%), mostly Western (*n* = 188, 69.1%), Eastern (*n* = 41, 15.1%) and Central (*n* = 29, 10.7%); only a few cases were from South America (*n* = 4, 1.5%), Asia and Oceania (*n* = 3, 1%). The most represented countries (not reported in Table) were Mali (19%), Ghana (14%), Ivory Coast (7%), Senegal (6%), Nigeria (5%), Tanzania (5%) and Ethiopia (4%). The countries of probable exposure are shown in detail in Fig. 1. The median elapsed time was 197 (67.5–526.5) days.

### Clinical presentation and laboratory characteristics

One hundred and forty-four (52.9%) patients presented at least one symptom suggestive of schistosomiasis (Table 2). The most common symptom was abdominal pain (36%), followed by macroscopic hematuria (11.3%), other genito-urinary symptoms (7.4%), rectal bleeding (1.5%), and cough (1.1%). None of the patients were diagnosed with Katayama syndrome. No significant differences were found in the symptom distribution between the two main groups.

**Table 2:** Clinical characteristics and laboratory results of the cohort and stratified by endemicity of provenience

Characteristic^a^	Entire cohort	Area of origin		*p*-value^b^
		Endemic	Non-endemic	
Total, *N* (%)	272	234 (86.0)	38 (14.0)	
Symptoms presence, *N* (%)	144 (52.9)	124 (52.9)	20 (52.6)	1
Abdominal pain	98 (36.0)	85 (36.3)	13 (34.2)	0.857
Genito-urinary	20 (7.4)	16 (6.8)	4 (10.5)	0.625
Macro-hematuria	25 (11.3)	22 (9.4)	3 (7.9)	1
Rectal bleeding	4 (1.5)	4 (1.7)	0 (0)	
Cough	3 (1.1)	3 (1.3)	0 (0)	
Positive microscopy, *N* (%)	103 (37.9)	98 (41.9)	5 (13.2)	< 0.001*
Ova in stools	50 (18.4)	48 (20.5)	2 (5.3)	0.020*
Ova in urine	52 (19.1)	49 (20.9)	3 (7.9)	0.070
Ova in biopsy	12 (4.4)	11 (4.7)	1 (2.6)	1
*Schistosoma* species, *N* (%)				
*S.haematobium*	49 (47.6)	46 (46.9)	3 (60.0)	0.102
*S.mansoni*	48 (46.6)	46 (46.9)	2 (40.0)	0.037*
Both	6 (5.8)	6 (6.2)	0 (0.0)	
Positive serology, *N* (%)	250 (91.9)	213 (91.0)	37 (97.4)	0.332
Increased IgE^c^, *N* (%)	169 (63.8)	159 (70.0)	10 (26.3)	< 0.001*
Blood eosinophilia, *N* (%)	130 (47.8)	115 (49.1)	15 (39.5)	0.297
Other helminths, *N* (%)	49 (22.8)	44 (18.8)	5 (13.2)	0.499
Abnormal Ultrasound, *N* (%)	124 (48.8)	110 (50.2)	14 (40.0)	0.280
Infection Site, *N* (%)				
IS	48 (17.9)	46 (20.0)	2 (5.4)	0.037*
HSS	13 (5.1)	13 (5.9)	0 (0)	
UGS	133 (48.9)	120 (51.3)	13 (34.2)	0.056
I	110 (43.8)	88 (40.6)	22 (64.7)	0.009*
Diagnosis Type, *N* (%)				
Confirmed	103 (37.9)	98 (41.9)	5 (13.2)	0.003*
Probable	165 (60.6)	133 (56.9)	32 (82.2)	
Suspected	4 (1.5)	3 (1.2)	1 (2.6)	

*Abbreviations: IS* Intestinal, *HSS* Hepato-splenic schistosomiasis, *UGS* Urogenital schistosomiasis, *I* Indeterminate

^a^Relative frequencies for each variable are obtained on the sub-population for which information was available

^b^Calculation of the *P*-value was done through different statistical tests. Namely, Kolmogorov-Smirnov test was used for testing the difference in the distribution of age and elapsed time, whereas Fisher’s exact test was used for all dichotomous variables. Finally, the Chi-Squared test was used to test the difference in the distribution among diagnoses

^c^Total IgE value equal or above the upper normal limit (≥120 IU/ml)

HIV infection (*n* = 4, 1.5%), chronic hepatitis B (*n* = 36, 13.2%), and HCV infection (*n* = 8, 2.9%) were exclusively found in the endemic group. One patient had the co-infection schistosomiasis/HCV/HBV.

Forty-nine subjects (22.8%) had other helminth infections, including both *S. stercoralis* diagnosed by immunodiagnostic method, as well as other intestinal helminths, diagnosed by parasitological method.

The median value of IgE and absolute eosinophil count were 304.5 IU/ml (IQR: 52.5–924.5) and 290 cell/μl (IQR: 160–540), respectively. Increased IgE was observed in 169 subjects (63.8%), while peripheral eosinophilia was present in 130 (47.8%) patients. The IgE level was more frequently raised in the endemic group (70 vs. 26.3%, *P* < 0.001), while no significant difference was found in the proportion of eosinophilia.

Schistosoma ova were detected in 103 subjects (37.9%): *S. haematobium* in 49 (47.6%), *S. mansoni* in 48 (46.6%) and both species in 6 subjects (5.8%). The ova were found in stools in 50 cases (18.4%), urine in 52 (19.1%), and tissue biopsy in 12 (4.4%), specifically: lungs (*n* = 2), liver (*n* = 2), uterus (*n* = 1), colon (*n* = 1) and appendix (*n* = 1). The immigrants were more likely to have ova detected than the non-endemic subjects (41.9 vs. 13.2%, *P* < 0.001).

Serology for schistosomiasis was positive in 250 subjects (91.9%) with no significant differences between the groups. IFAT serology was used in 163 cases (59.9%), while 109 patients (40.1%) were tested by ELISA test.

Eighteen of 103 patients (17.5%) with schistosoma ova identified by microscopy were negative by serology. Eleven of these 18 patients (61.1%) had *S. haematobium* infection.

Urine CCA test was found positive in 14 of 61 (23%) patients evaluated with this test.

### Ultrasound findings and patterns

**Fig. 2:**
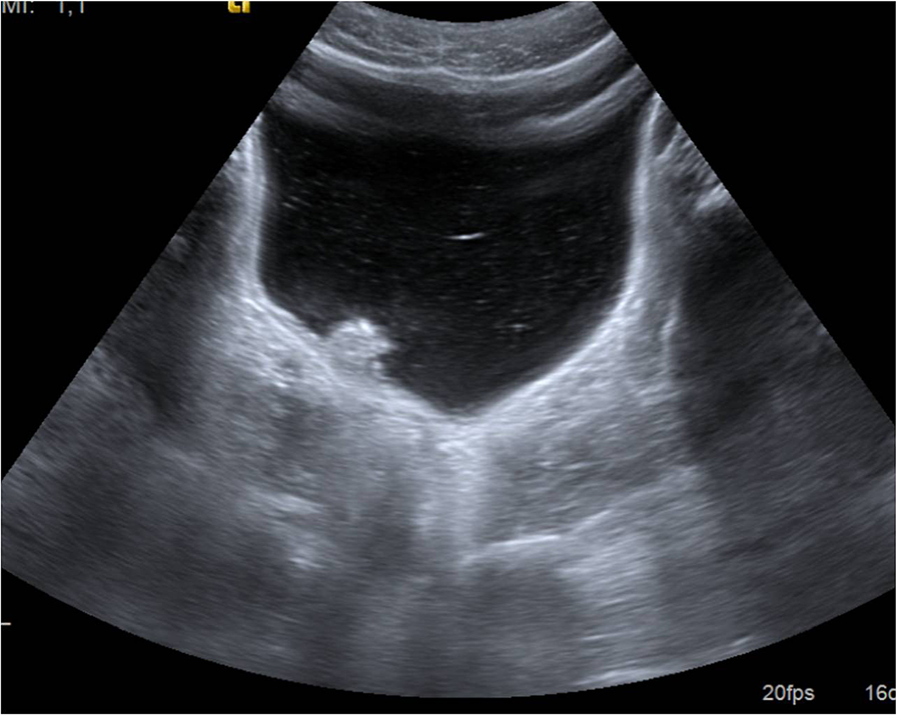
Pseudopolyp of the urinary bladder in an immigrant with *S. haematobium* infection

Ultrasounds showed abnormalities in 124 of 254 subjects examined (48.8%), including: liver enlargement (*n* = 64, 25.2%), spleen enlargement (*n* = 35,13.8%), cirrhosis (*n* = 8, 3.2%), bladder lesions (*n* = 22, 8.7%) (Fig. 2), thick bladder wall (*n* = 45, 17.7%), portal hypertension (*n* = 6, 2.4%) and periportal thickening/fibrosis (*n* = 6, 2.4%). In summary, 52 (20.5%) patients’ ultrasound findings were compatible with the UG pattern and 13 (5%) with the HS pattern, without differences between the groups (data not shown).

Thirty-six of 103 (34.9%) patients with schistosomiasis confirmed by microscopy had an abnormal ultrasound pattern: 30 (29.1%) UG, three (2.9%) HS, and three patients (2.9%) both patterns. Differently, in patients with schistosomiasis with positive serology test without evidence of ova, 21 (12.7%) had ultrasound alterations: five (3%) HS and 16 (9.7%) UG pattern.

Globally, rates of ultrasound abnormalities found in confirmed schistosomiasis and in patients with positive serology only (34.9% vs. 12.7%) were statistically different (*P* < 0.001).

### Case definition

According to the clinical features, the patients were classified as follows: 133 (48.9%) with UGS, 48 (17.9%) with IS, 13 (5.1%) with HSS and 110 (43.8%) with indeterminate schistosomiasis. The diagnosis was confirmed in 103 cases (37.9%), probable in 165 (60.6%), and possible in four (1.5%) (Table 2).

### Univariate and multivariate analyses

Table 3 shows the results of the univariate and multivariate analyses on the association between microscopic ova identification and demographic characteristics, as well as laboratory/ultrasound findings. Younger age, abnormal ultrasound findings and blood eosinophilia were significantly associated with a positive microscopy (*OR* = 0.94, 95% *CI*: 0.91–0.97; *OR* = 2.12, 95% *CI*: 1.15–3.96; *OR* = 1.98, 95% *CI*: 1.03–3.83, respectively).

**Table 3:** Crude and adjusted *OR*s for the association between patients’ characteristics and the risk of detection of ova in any specimen^a^

Covariate^b^	Ova present	Ova absent	Crude *OR*	Adjusted *OR* (95% *CI*)^c^
Age	26 (21–32.5)	34 (27–44)	0.93 (0.90–0.95)	0.94 (0.91–0.97)
Male	91.3%	71.0%	4.22 (1.92–10.31)	2.04 (0.78–5.81)
Elapsed Time	184 (56–328)	209 (71–657)	0.99 (0.99–0.99)	1.00 (0.99–1.01)
Other Parasites	18.4%	17.5%	1.06 (0.53–2.11)	0.79 (0.36–1.70)
Endemics	95.2%	80.7%	4.66 (1.72–15.87)	2.08 (0.64–8.10)
Abnormal ultrasound	61.0%	41.0%	2.23 (1.30–3.89)	2.12 (1.15–3.96)
Symptoms presence	59.2%	50.0%	1.45 (0.86–2.46)	1.82 (0.98–3.42)
Increased IgE^d^	80.0%	53.7%	3.43 (1.87–6.49)	1.20 (0.53–2.72)
Blood eosinophilia	64.0%	37.3%	2.98 (1.74–5.15)	1.98 (1.03–3.83)

*Abbreviations: OR* odds ratio, *CI* confidence interval

^a^Due to the heterogeneity of provenience of patients, covariates such as exposure time could not be included in models

^b^Relative frequencies are presented here, whereas the median (interquartile range) is reported for Age and Elapsed Time

^c^Adjusted for every other covariate through a multivariate logistic regression model

^d^Total IgE value equal or above the upper normal limit (≥ 120 IU/ml)

## Discussion

Sentinel surveillance data for imported schistosomiasis in Europe has been available since 1997 through the European Network for Tropical Medicine and Travel Health (TropNet) [32, 36] and the GeoSentinel Surveillance Network [34]. Moreover, a few observational studies were performed by individual tropical centres in Munich (Germany) [30], Barcelona (Spain) [31], Amsterdam (the Netherlands) [33] and London (United Kingdom) [35].

Most of our cases (96%) were imported from the African continent, primarily West Africa, similarly to all studies mentioned above [30–36]. A peculiarity of our study is the high proportion of migrants (86%), of whom more than two-thirds were young male refugees and asylum seekers (all from Africa), in contrast with travellers and expatriates. The recent high flow of asylum seekers from Africa to Europe (and particularly to Italy) largely explains the demographic characteristics observed [17].

Previous screening studies conducted on migrant populations in Europe found a questionably low prevalence of schistosomiasis, probably depending on the diagnostic tools used [19, 21, 23–25]. Indeed, only about 40% of our patients included in the endemic group had a diagnosis of confirmed schistosomiasis, and therefore, had we relied on ova identification only, the number of cases diagnosed would have been much lower. On the other hand, we have recently shown that the sensitivity of ova detection is comparatively low [26]. It might be argued that a number of false positive cases may be comprised among those classified as “probable” on the basis of serology. However, the specificity of serology is high, and so is that of CCA test [26] hence the number of false positive cases (if any) is probably limited.

Almost half of the patients were asymptomatic. These data concur with other studies, reporting proportions ranging from 36 to 50%, depending on the population analysed [36]. Coltart et al. described a more frequently asymptomatic presentation in the non-endemic group [35]: the most frequent symptom reported was abdominal pain and, although almost half of the cases were considered UGS, only 11% of the patients reported macroscopic haematuria. Differently from Coltart’s work, several studies showed differences in clinical presentation between the endemic and the non-endemic groups. Lingscheid et al. found that Europeans presented more frequently fever and musculoskeletal symptoms, whereas the non-endemic group complained more often of haematuria, bloody diarrhoea and weight loss compatible with chronic schistosomiasis [36]. The high proportion of migrants in our series (86%, contrarily to other series in which the tourists represented the vast majority), along with the relatively long time elapsed from exposure to presentation to hospital of both groups (7 months in endemic and 4 months in non-endemic), probably accounts for the absence of febrile cases compatible with acute schistosomiasis [33, 34, 51]. Of note, a high proportion of cases had already developed symptoms/signs compatible with chronic schistosomiasis, and the treatment probably permitted to prevent life-threatening complications. However, almost half of the cases were asymptomatic, so this parasitic infection would have never be detected in this group if not systematically searched, based on epidemiological criteria.

Eosinophilia was present only in the 47.8% of the cases. Interestingly, a higher proportion (63.8%) of subjects with increased IgE value was found, consistent with a recent study performed in an endemic area for *S. mansoni* [52]. In our study, some differences were found in the laboratory findings of the two main groups. In particular, a significantly higher proportion of patients with raised IgE was found in the endemic group.

The proportion of patients with positive serology was 92%, whereas a confirmed parasitological diagnosis was achieved in 38% of patients; among the latter, 18 cases had negative serology. Worthy to note, the immunodiagnostic method was changed from IFAT to ELISA during the course of the study due to availability constraints. However, different studies showed similar sensitivity for the two tests, that is higher for *S. mansoni* infection [53, 54]. Indeed, in our study, most of the 18 confirmed cases with negative serology (61%) had *S. haematobium* infection, for which serology has sensitivity ranging from 21.4 to 71.4%. [53].

In line with the literature, we considered only microscopy as gold standard for diagnosis [1]. The accuracy of CCA test for *S.mansoni* is not optimal, especially in case of urinary tract infections, hematuria, pregnancy and in young children [55]. Furthermore, the test was available only in the late stage of our study, and few results were available.

Abnormal US findings were present in about half of the study subjects. Hepatomegaly or splenomegaly was reported in 25.3 and 13.8% of cases, respectively, but of course this can be due to causes other than schistosomiasis. Therefore, abdominal organomegaly was not considered a sufficient criterion to define hepatosplenic schistosomiasis in our cohort. We then probably underestimated the proportion of HSS, especially in the early stage, which is most susceptible to regression after treatment.

Differently, thick bladder wall and bladder lesions, both considered signs of *S. haematobium* infection, were found in 17.7 and 8.7% of the cases, respectively, and permitted to identify 20.5% patients with UGS.

No cases of liver or bladder cancer were found in our study.

The higher rate of US abnormalities found in patients with positive microscopy compared with patients with positive serology only is not surprising. Serology can detect a higher number of infections of low intensity, difficult to diagnose with stool/urine microscopy. Presumably, these are stages of infection that may still cause organ damage. Nevertheless, we cannot exclude some false positive results of serology, especially with regard to possible previous infections and treatments [56]. Assessing the efficacy (adequate dosage and duration) of previous treatments is challenging, and so is evaluating possible re-exposures occurred after treatment, particularly for migrants at first arrival. In consideration of the short-term treatment’s high tolerability, it is worth treating subjects who are positive to serology only.

The association of ova identification with abnormal US findings and with blood eosinophilia (Table 3) is interesting, as it seems to suggest a correlation of these findings with the parasitic load. Neither finding, however, can be considered a good predictor or excluder of this infection, thus their clinical use is limited to potentially raising the index of suspicion, particularly in settings where accurate parasitological methods are not often accessible, and only a few (if any) immunodiagnostic methods are available.

On the other hand, we did not find any significant association between symptoms and ova detection, a factor which further supports the need for an active screening.

The association between younger age and positive microscopy confirms data already reported in the literature. Precisely, in endemic settings, people are often exposed to repeated infections during childhood and adolescence, and gradually develop an efficient immune response through time [52].

Our study has limitations, mainly due to its retrospective design. Firstly, we were not able to assess ova intensity in cases confirmed by microscopy, because these data had not been collected. Secondly, abdomen US had been performed following standard, clinical routine, which means that we might have missed initial forms of HSS or UGS. Finally, as mentioned above, the serological test for schistosomiasis was changed during the study period.

## Conclusions

European clinicians need to be alerted about possible cases of schistosomiasis in subjects from endemic countries, particularly from sub-Saharan Africa.

The gold standard test permitted to diagnose only one-third of cases. Clinical and diagnostic presentation can be silent in patients with schistosomiasis. Symptoms, eosinophilia and abnormal US findings may raise the index of suspicion both in patients born in endemic and in non-endemic countries. The combination of different diagnostic tools may facilitate the diagnosis and allow the treatment of an infection that can lead to severe complications.

## Additional file

Additional file 1: Multilingual abstracts in the five official working languages of the United Nations. (PDF 537 kb)

## Abbreviations

AEC: Absolute eosinophils count
CCA: Cathodic circulating schistosomal antigen
*CI*: Confidence interval
CTD: Centre for Tropical Diseases
ELISA: Enzyme-linked immunosorbent assay
HS: Hepatosplenic
HSS: Hepatosplenic schistosomiasis
IFAT: Indirect immunofluorescence
IQR: Inter-quartile range
IS: Intestinal schistosomiasis
NTD: Neglected tropical diseases
*OR*: Odds ratio
UG: Urogenital
UGS: Urogenital schistosomiasis
VFR: Visiting friends or relatives
