# Supplementary material for: Schistosomiasis in immigrants, refugees and travellers in an Italian referral centre for tropical diseases
Source: Infect Dis Poverty. 2018 Jun 16;7:55. doi: 10.1186/s40249-018-0440-5 (PMC6004084; doi:10.1186/s40249-018-0440-5)
Supplement: Supplementary file 1 — Multilingual abstracts in the five official working languages of the United Nations. (PDF 537 kb) [file 40249_2018_440_MOESM1_ESM.pdf]

Translation of the abstract into the five official working languages of the United Nations

داء البلهارسيا لدى المهاجرين واللاجئين والمسافرين في أحد مراكز الأحالة الإيطالية للأمراض الاستوائية

فالنتينا ماركيزي، آنا بيلترامي، أندريا أنغيين، جيرالدو بادونا مونتيرو، جيوفاني جيورلي، فرانسيكا بيراندين، دورا بونفراتي وزينو بيسوفي

الملخص

الخلفية: داء البلهارسيا هو أحد أخطر الأمراض الاستوائية التي لا تحظى بالاهتمام الكافي. يمكن للعدوى المزمنة بهذا المرض، إذا لم يتم التعرف على أعراضها وعلاجها، أن تؤدي إلى حدوث مضاعفات لا يمكن معالجتها.

الوسائل: دراسة مراقبة استعادية تهدف إلى وصف التاريخ السريري والنتائج المعملية والسمات الظاهرة في الصور التشخيصية لحالات البلهارسيا الخارجية التي تم تشخيصها في مركز الأمراض الاستوائية بمستشفى ساكرو كوري دون كالابريا بنيغرار فيرونا في إيطاليا، في الفترة من 2010 إلى 2014. هدف دراستنا هو تقييم الاختلافات بين المهاجرين/الأصدقاء والأقارب الزائرين من الدول المستوطنة بالمرض (فئة مواطني الدول المستوطنة) والمغتربين/المسافرين (فئة مواطني دول غير مستوطنة)، وذلك من حيث الخصائص الديموغرافية والسمات السريرية والبيانات المعملية ونتائج التصوير بالموجات فوق الصوتية.

النتائج: تم جمع 272 مريضًا إجمالًا: 234 في فئة مواطني الدول المستوطنة و38 في فئة مواطني الدول غير المستوطنة. أصيب أغلب المرضى بمرض البلهارسيا في قارة إفريقيا (97.4%). أبلغ 52.9% من المرضى بظهور أعراض، وكان أكثر الأعراض تكرارًا هي ألم البطن (36%) والبول الدموي العياني (11.3%) والأعراض البولية التناسلية (7.4%). لوحظت زيادة الغلوبين المناعي IgE وكثرة اليوزينيات لدى 169 (63.8%) و130 (47.8%) من المرضى، على التوالي. كانت نسبة النتائج الإيجابية لفحوصات مصل الدم 250/272 (91.9%).كانت نتيجة اختبار المستضد الكاثودي الدوراني (CCA) المستخدم للكشف عن طفيل الشيستوسوما مانسوني إيجابية لدى 14/61 مريض (23%). كانت نسبة المرضى المصابين بالعدوى، بعد الفحص المجهري، 103/272 (37.9%). كانت فصائل الشيستوسوما التي تم العثور عليها هي شيستوسوما هيماتوبيوم (47.6%)، أو شيستوسوما مانسوني (46.6%) أو كلاهما (5.8%). صُنفت حالات داء البلهارسيا إلى حالات مؤكدة لدى 103 (37.9%) ومحتملة لدى 165 (60.6%) ومشتبه بها لدى 4 (1.5%) من الحالات، وذلك باستخدام السمات السريرية والبيانات المعملية ونتائج الفحوصات بالموجات فوق الصوتية. صُنفت العدوى كذلك حسب الأعضاء المصابة إلى: عدوى أمعائية (17.9%) وكبدية طحالية (5.1%) وبولية تناسلية (48.9%) وغير محددة (43.8%).

أبرز التحليل المقارِن لمرضى الدول المستوطنة وغير المستوطنة بالمرض وجود اختلافات حسب النوع والعمر. كان معدل التعرف على بويضات الطفيليات لدى مرضى الدول المستوطنة بالمرض أعلى (41.9% مقابل 13.2%، P < 0.001) وكانت نسبة الغلوبولين المناعي IgE لديهم أعلى (70% مقابل 26.3%، P < 0.001) مقارنة بمرضى الدول غير المستوطنة بالمرض. أظهرت التحليلات ذات العوامل المتغيرة المتعددة ارتباط صغر السن واختلال نتائج الفحوصات بالموجات فوق الصوتية وكثرة اليوزينيات بالدم ارتباطًا كبيرًا بوجود نتائج إيجابية للفحوصات المجهرية (OR = 0.94، OR = 2.12، OR = 1.98، على التوالي).

النتائج: كانت أعراض المرض وكثرة اليوزينيات واختلال نتائج الفحوصات بالموجات فوق الصوتية موجودة لدى نصف المرضى تقريبًا، دون اختلافات بين الفئتين. كانت نتائج فحوصات مصل الدم إيجابية لدى العديد من المرضى، ولكن كانت الفحوصات المجهرية لديهم سالبة ما يشير إلى احتمال إساءة تشخيص الإصابة بداء البلهارسيا. يمكن لاستخدام مجموعة من الأدوات التشخيصية أن يجعل التشخيص أكثر سهولة.

Translated from English version into Arabic by Salma Anwar and Farah Dar, through

TRANSLATORS WITHOUT BORDERS

意大利热带病转诊中心的移民、难民和旅游人群中的血吸虫病情况

Valentina Marchese, Anna Beltrame, Andrea Angheben, Geraldo Badona Monteiro, Giovanni Giorli, Francesca Perandin, Dora Buonfrate and Zeno Bisoffi

摘要：

引言：血吸虫病是一种重要的被忽视热带病。如果患者未能及时发现并就诊，长期感染将导致不可逆的并发症。

方法：本回顾性观察研究描述分析了 2010–2014 年意大利维罗那内格拉尔圣心唐卡拉布里亚医院（Sacro Cuore Don Calabria Hospital）热带病中心诊断的输入性血吸虫病患者的病史、实验室检查结果和影像学表现。本研究旨在评估来自血吸虫病流行区国家的移民、探亲访友者（VFR）（流行组）和侨民或旅游者（非流行组）之间在人口学特征、临床表现、实验室数据和超声结果方面的差异。

结果：本研究共检索 272 例患者资料，其中 234 例来自流行地区，38 例来自非流行地区。大部分患者(97.4%)在非洲感染血吸虫病。据统计，52.9%的患者出现症状，最常报道的为腹痛(36%)、肉眼血尿(11.3%)和泌尿道症状(7.4%)。169 例（63.8%）和 130 例（47.8%）患者的 IgE 和血液嗜酸性粒细胞增多。血清学阳性比例为 250/272(91.9%)。14 (14/61，23%)例患者的曼氏血吸虫循环趋阴极抗原 CCA 阳性。103 (103/272，37.9%)例患者镜检阳性。感染血吸虫的种类分别为埃及血吸虫(47.6%)、曼氏血吸虫(46.6%)和二者共感染(5.8%)。根据临床表现、实验室检查和超声结果，诊断情况可分为确诊 103 例(37.9%)、临床诊断 165 例(60.6%)和疑似 4 例(1.5%)。根据受累器官进一步分为：肠感染(17.9%)、肝脾感染(5.1%)、泌尿生殖道感染(48.9%)和未确定(43.8%)。

通过对比分析流行组和非流行组发现两组间性别和年龄存在差异。同非流行组相比，流行组更易检出虫卵(41.9% vs. 13.2%，$P < 0.001$)且 IgE 更高(70% vs. 26.3%，$P < 0.001$)。多变量分析表明，低龄、超声异常和血中嗜酸性粒细胞增多同镜检阳性显著相关(*OR* 值分别为 0.94、2.12、1.98)。

结论：约半数患者有临床症状、嗜酸性粒细胞增多和超声结果异常，但这些在两组之间没有差异。多数患者血清学检查阳性，但镜检阴性，表明可能存在误诊。综合运用多种诊断工具或可更有效促进正确诊断疾病。

Translated from English version into Chinese by Peng Song, edited by Jin Chen

CDC CHINESE CENTER FOR DISEASE CONTROL AND PREVENTION
NATIONAL INSTITUTE OF PARASITIC DISEASES

La schistosomiase parmi les migrants, réfugiés et voyageurs vus dans un centre de référence italien pour les maladies tropicales

Valentina Marchese, Anna Beltrame, Andrea Angheben, Geraldo Badona Monteiro, Giovanni Giorli, Francesca Perandin, Dora Buonfrate et Zeno Bisoffi

Résumé

Contexte : La schistosomiase est une des maladies tropicales négligées les plus importantes. Si elle n'est pas reconnue ni traitée, l'infestation chronique peut entraîner des complications irréversibles.
Méthodes : Étude observationnelle rétrospective décrivant l'historique clinique, les résultats de laboratoire et les résultats d'imagerie de cas de schistosomiase importée diagnostiqués au Centre des maladies tropicales de l'Hôpital Sacro Cuore Don Calabria à Negrar (Vérone), en Italie, de 2010 à 2014. Le but de notre étude était d'évaluer les différences de caractéristiques démographiques, de présentation clinique, de paramètres de laboratoire et de résultats d'échographies entre des immigrés et proches en visite provenant de pays d'endémie (groupe d'endémie) et des expatriés ou voyageurs (groupe sans endémie).
Résultats : Nous avons trouvé au total 272 patients, 234 dans le groupe d'endémie et 38 dans le groupe sans endémie. La plupart des patients avaient contracté la schistosomiase en Afrique (97,4 %) et 52,9 % signalaient des symptômes, notamment des douleurs abdominales (36 %), une hématurie microscopique (11,3 %) et des symptômes génito-urinaires. Des taux élevés d'IgE et une éosinophilie sanguine ont été observés chez respectivement 169 (63,8 %) et 130 (47,8 %) patients. Le taux de séropositivité était de 250 patients sur 272 (91,9 %).Le test ACC (antigène cathodique circulant) pour *Schistosoma mansoni* était positif chez 14 patients sur 61 (soit 23 %). L'examen au microscope a fait apparaître une infestation chez 103 sujets sur 272 (37,9 %). Les espèces de schistosomes découvertes étaient *S. haematobium* (47,6 %), *S. mansoni*(46,6 %) ou les deux (5,8 %). La schistosomiase a été considérée comme confirmée chez 103 patients (37,9 %), probable chez 165 (60,6 %) et suspectée chez 4 (1,5 %), sur la base de la présentation clinique, des données obtenues en laboratoire et des résultats d'échographie. Selon les organes affectés, elle a été classifiée en atteinte intestinale (17,9 %), hépatosplénique (5,1 %), urogénitale (48,9 %) et indéterminée (43,8 %).
L'analyse comparative des patients provenant des régions d'endémie et sans endémie a mis en évidence des différences en termes d'âge et de sexe. L'identification d'œufs était plus fréquente chez les patients du groupe d'endémie (41,9 % contre 13,2 %, $P < 0,001$), qui présentaient également une élévation des IgE plus fréquente (70 % contre 26,3 %, $P < 0,001$) que dans le groupe sans endémie. Les analyses multivariées ont montré que chez les patients jeunes, des anomalies à l'échographie et une éosinophilie étaient significativement associés à une microscopie positive ($OR = 0,94$, $OR = 2,12$, $OR = 1,98$, respectivement).
Conclusions : La moitié environ des patients présentaient des symptômes, une éosinophilie et des anomalies à l'échographie, sans différences entre les groupes. De nombreux patients avaient une sérologie positive mais une microscopie négative, ce qui pourrait indiquer des schistosomiases mal diagnostiquées. La combinaison de différents outils de diagnostic pourrait faciliter le diagnostic.

Translated from English version into French by Iris Soliman and Suzanne Assenat, through

TRANSLATORS WITHOUT BORDERS

Шистосомоз у иммигрантов, беженцев и путешественников в итальянском координационном центре по изучению тропических заболеваний

Валентина Марчезе, Анна Белтраме, Андреа Ангхебен, Жеральдо Бадона Монтейро, Джованни Джиорли, Франческа Перандин, Дора Буонфрате и Зено Бисоффи

Аннотация

Справочная информация: Шистосомоз является одним из самых важных забытых тропических болезней. Если заболевание вовремя не распознать и не начать лечение, хроническая инфекция может привести к необратимым осложнениям.

Методы: Ретроспективное исследование методом наблюдения, направленное на описание клинической истории, лабораторных результатов и визуальную презентацию завезённого шистосомоза, диагностированного в Центре по изучению тропических заболеваний, Госпиталь «Сакро Куоре Дон Калабрия», ул. Неграр, Верона, Италия, в период с 2010 по 2014 гг. Цель данного исследования заключалась в оценке различий в демографических характеристиках, клиническом проявлении, лабораторных данных и результатах ультразвуковых исследований у иммигрантов/посетителей друзей и родственников (ПДР) из эндемических стран (эндемической группы) и у экспатриантов/путешественников (неэндемическая группа).

Результаты:В исследовании приняло участие в общей сложности 272 пациента: 234 человека из эндемической группы и 38 человек из неэндемической группы. Большинство пациентов приобрели шистосомоз в Африке (97,4%). О симптомах сообщили 52,9% пациентов; чаще всего заявляли об абдоминальной боли (36%), макрогематурии (11,3%) и мочеполовых симптомах (7,4%). Повышенный уровень иммуноглобулина Е и эозинофилов крови наблюдался у 169 (63,8%) и 130 (47,8%) пациентов, соответственно. Доля положительных результатов серологических исследований составила 250/272 (91,9%).Результаты тестирования на циркулирующий катодный антиген (CCA) по *шистосоме Мансони*оказались положительными у 14/61 пациентов (23%). При микроскопическом исследовании количество инфицированных пациентов составило 103/272 (37,9%). Выявленными видами шистосомы были *шистосома гематобиум* (47,6%), *шистосома Мансони* (46,6%) или оба вида (5,8%). На основе клинических проявлений, лабораторных данных и результатов ультразвуковых исследований шистосомоз был классифицирован как подтвержденный в 103 (37,9%), вероятный в 165 (60,6%) и предполагаемый в 4 (1,5%) случаях. Затем инфекцию классифицировали с точки зрения затронутого органа: кишечная (17,9%), гепатолиенальная (5,1%), урогенитальная (48,9%) и недифференцированная (43,8%).

Сравнительный анализ данных эндемических и неэндемических пациентов выявил различия в зависимости от пола и возраста. У эндемических пациентов чаще наблюдалось овальбумин-специфичное проявление (41,9% по сравнению с 13,2% P P<0,001), и повышенный имуноглобулин Е (70% по сравнению с 26,3%, P < 0,001), чем у не эндемических. Многофакторный анализ показал, что более молодой возраст, аномальные результаты ультразвуковых исследований и уровень эозинофилов в крови в значительной степени связаны с положительными результатами микроскопических исследований (*коэффициент риска OR* =0,94; *OR*=2,12; *OR*=1,98, соответственно).

Выводы: Симптомы, эозинофилия и аномальные результаты ультразвуковых исследований наблюдались примерно у половины пациентов, различий между группами не наблюдалось.

У многих пациентов были получены положительные результаты серологических исследований, но отрицательные при микроскопических исследованиях, что указывает на возможную неверную диагностику шистосомоза. Сочетание диагностических методов может способствовать постановке диагноза.

Translated from English version into Russian by Helga_Sh and Liudmila Tomanek, through

Esquistosomiasis en inmigrantes, refugiados y viajeros en un centro de referencia italiano para Enfermedades Tropicales

Valentina Marchese, Anna Beltrame, Andrea Angheben, Geraldo Badona Monteiro, Giovanni Giorli, Francesca Perandin, Dora Buonfrate y Zeno Bisoffi

Resumen

Trabajo previo: La esquistosomiasis es una de las enfermedades tropicales desatendidas más importantes. Si no se reconoce ni se trata, la infección crónica puede derivar en complicaciones irreversibles.

Métodos: Estudio observacional retrospectivo destinado a describir la historia clínica, los hallazgos de laboratorio y la presentación de imágenes de la esquistosomiasis importada diagnosticada en el Centro de Enfermedades Tropicales, Hospital Sacro Cuore Don Calabria de Negrar, Verona, Italia, de 2010 a 2014. El objetivo de nuestro estudio fue evaluar las diferencias en las características demográficas, presentación clínica, datos de laboratorio y hallazgos de ultrasonido entre inmigrantes/amigos visitantes y familiares (AVF) de países endémicos (grupo endémico) y expatriados/viajeros (grupo no endémico).

Resultados: Un total de 272 pacientes se recuperaron: 234 en el grupo endémico y 38 en el no endémico. La mayoría de los pacientes adquirieron esquistosomiasis en África (97.4%). Los síntomas fueron comunicados por el 52.9% de los pacientes; los síntomas más frecuentes que describieron los pacientes fueron el dolor abdominal (36%), la hematuria macroscópica (11.3%) y los síntomas genitourinarios (7.4%) Se observó un incremento de IgE y eosinofilia sanguínea en 169 (63.8%) y 130 (47.8%) pacientes, respectivamente. La proporción de serología positiva fue de 250 a 272 (91.9%).El Antígeno Catódico Circulante CCA para *Schistosoma mansoni* fue positivo en 14 de 61 personas (23%). En microscopía, los sujetos infectados fueron 103 de 272 (37.9%). Las especies de Schistosoma encontradas fueron *S. haematobium* (47.6%), *S. mansoni* (46.6%) o las dos (5.8%). La esquistosomiasis fue clasificada como confirmada en 103 (37,9%), probable en 165 (60,6%) y se sospechó en 4 (1,5%) casos usando presentación clínica, datos de laboratorio y hallazgos de ultrasonido. La infección se clasificó además basada en la afectación del órgano: intestinal (17.9%), hepatoesplénica (5.1%), urogenital (48.9%) e indeterminada (43.8%).

El análisis comparativo entre pacientes endémicos y no endémicos resaltó las diferencias en sexo y edad. Los pacientes endémicos tenían una identificación más frecuente de los óvulos (41.9% vs.

13.2%, $P < 0.001$) y IgE más elevada (70% vs. 26.3%, $P < 0.001$) en comparación con los no endémicos. Los análisis multivariados mostraron que en edad másjoven, los hallazgos eco gráficos anormales y la eosinofilia sanguínea se asociaron significativamente con micro positivoscopy (*O* = 0.94, *O* = 2.12, *O* = 1.98, respectivamente).
Conclusiones: Los síntomas, la eosinofilia y los hallazgos de ultrasonido anormales estuvieron presentes en aproximadamente la mitad de los pacientes, sin diferencias entre los grupos. Muchos pacientes tenían serología positiva pero microscopia negativa, lo que indica que la esquistosomiasis podría ser mal diagnosticada. Una combinación de herramientas de diagnóstico puede facilitar el diagnóstico.

Translated from English version into Spanish by Ignacio Montalvo and Marc Gonzalez, through

TRANSLATORS
WITHOUT BORDERS
